# Combined Dietary Supplementation with *Bacillus subtilis* DSM 29784 and a Phytogenic Blend Modulates the Microbiome–Gut–Bone Axis in Broiler Chickens

**DOI:** 10.3390/ani16213333

**Published:** 2026-10-22

**Authors:** Nanthawath Saikhwan, Damrongsak Faroongsarng, Yuwares Ruangpanit, Autchariya Thodsapol, Konkawat Rassmidatta, Tim Goossens, Nuria Vieco-Saiz, Damien P. Prévéraud, Yongyuth Theapparat

**Affiliations:** 1 Center of Excellence in Functional Foods and Gastronomy, Faculty of Agro-Industry, Prince of Songkla University, Hat Yai 90110, Thailand; 2 Research Center of Microbiome Systemic Engineering, Prince of Songkla University, Hat Yai 90110, Thailand; 3 Department of Pharmaceutical Technology, Faculty of Pharmaceutical Science, Prince of Songkla University, Hat Yai 90110, Thailand; 4 Department of Animal Science, Faculty of Agriculture at Kamphaeng Sean, Kasetsart University, Kamphang Saen Campus, Kamphaeng Saen 73140, Thailand; 5 Center for Genomics and Bioinformatics Research, Faculty of Science, Prince of Songkla University, Hat Yai 90110, Thailand; 6 Adisseo, Gentse Baan 66/206, 9100 Sint-Niklaas, Belgium; tim.goossens@adisseo.com; 7 European Laboratory of Innovation, Science & Expertise (ELISE), Adisseo France S.A.S, 20 rue Prosper Monnet, 69190 Saint Fons, France; 8 Adisseo France S.A.S., 92160 Antony, France

**Keywords:** broiler chicken, *Bacillus subtilis* DSM 29784, phytogenic feed additive, gut microbiome, osteoimmunology

## Abstract

The microbiome–gut–bone axis may influence skeletal physiology in fast-growing broilers. This study evaluated dietary *Bacillus subtilis* DSM 29784 combined with a phytogenic blend (BsP) in 560 male Ross 308 broilers assigned to control or BsP diets (eight pens/treatment; 35 birds/pen) for 42 days. One clinically normal bird per pen was sampled on days 21, 35, and 42. BsP increased final body weight and cumulative gain and reduced the overall feed conversion ratio (*p* < 0.05). Intestinal barrier-associated gene expression increased, several ileal inflammatory marker transcripts decreased, and mineral-handling-related gene expression and serum/femoral mineral concentrations were higher at selected ages. Cecal 16S rRNA gene profiles showed differences in diversity and taxonomic composition. PICRUSt2 identified differences in predicted microbial functional potential, not measured pathway activity or metabolite production. Femoral inflammatory and osteogenic gene expression responses varied with age. Exploratory networks summarized microbiome–gut–bone associations at days 21, 35, and 42 without establishing causality or within-bird temporal change. BsP was associated with improved growth and altered intestinal and bone-related indicators, but clinical BCO prevention was not established. Future factorial studies should include direct bone quality measurements, standardized clinical and lesion assessments, and targeted microbial metabolite measurements.

## Simple Summary

Fast-growing broiler chickens can develop leg and bone problems that affect welfare and production. The intestine may influence skeletal physiology through nutrient absorption, inflammation, and signals associated with the gut microbial community. We tested a diet containing the probiotic bacterium *Bacillus subtilis* DSM 29784 together with a phytogenic blend. Supplemented birds had better final growth and feed efficiency, changes in intestinal barrier-associated and inflammatory gene expression, and higher blood and femoral mineral concentrations at selected ages. The diet also altered intestinal bacterial profiles and reduced DNA signals from some bacteria associated with bone infections. Laboratory assessments were performed in birds without overt clinical abnormalities. These findings concern microbial, molecular, and mineral responses; they do not demonstrate stronger bones or prevention of bacterial chondronecrosis with osteomyelitis. They support further evaluation of the combined dietary strategy using direct bone quality measurements and systematic clinical assessments.

## 1. Introduction

Skeletal disorders and leg abnormalities remain important welfare and production concerns in modern broiler systems. Rapid growth and high breast-muscle accretion increase mechanical loading on the developing skeleton and can contribute to conditions such as tibial dyschondroplasia, valgus–varus deformity, femoral head necrosis, and bacterial chondronecrosis with osteomyelitis (BCO) [1,2,3,4,5]. These disorders impair mobility and productivity and can predispose birds to secondary complications. Nutritional strategies that support intestinal health, mineral utilization, and skeletal development may therefore provide a complementary approach to reducing leg health risk in fast-growing broilers.

The gut microbiota contributes to host nutrient metabolism, immune regulation, barrier function, and systemic signaling, all of which can influence bone homeostasis [3,6,7,8,9,10]. Microbial metabolites, including short-chain fatty acids, vitamins, and other bioactive compounds, may affect osteoblast and osteoclast activity, whereas intestinal dysbiosis and barrier dysfunction can increase inflammatory exposure and impair calcium and phosphate utilization [10,11,12,13]. Accordingly, the microbiome–gut–bone axis provides a useful framework for examining how nutritional modulation of intestinal ecology may be linked with skeletal physiology in poultry.

Probiotics, particularly Bacillus-based strains, have been used in poultry to improve intestinal function, microbial balance, and feed efficiency [14,15]. *Bacillus subtilis* DSM 29784 has previously been associated with improved broiler performance, intestinal morphology, and enrichment of butyrate-producing bacteria [14]. Probiotic supplementation has also been linked with changes in bone mineralization and osteogenic activity [16,17]. However, relatively few studies have integrated microbial, intestinal, inflammatory, mineral-transport, and bone-related responses within the same experimental framework.

Phytogenic feed additives, including garlic-derived preparations, have antimicrobial, antioxidant, and immunomodulatory properties [18,19,20]. Garlic essential oil has also been evaluated at 200 mg/kg feed in broilers under environmental heat stress [21]. Organosulfur compounds and essential oil components can influence intestinal barrier integrity and microbial ecology, and combinations with probiotics have been explored as complementary nutritional approaches in other systems [22,23]. Phytogenic diets have also been investigated in aquaculture [24], providing cross-species context rather than evidence of efficacy of the present formulation in broilers. A combined *B. subtilis* DSM 29784–phytogenic intervention may therefore affect multiple biological levels relevant to the microbiome–gut–bone axis.

The specific knowledge gap is whether the combined *B. subtilis* DSM 29784–phytogenic intervention is associated with coordinated intestinal, microbial, mineral, and femoral responses across growth stages in broilers. We hypothesized that, compared with the basal diet, BsP would improve growth performance and be accompanied by higher barrier-associated and osteogenic marker expression, lower inflammatory marker expression, altered cecal microbial profiles, and differences in mineral status. Accordingly, the study jointly evaluated these outcomes and targeted BCO-associated bacterial DNA signals, with laboratory assessments in clinically normal birds. Age-specific networks at days 21, 35, and 42 were used to describe cross-domain covariation. The study evaluated biological responses that could motivate future preventive trials, rather than treatment of established disease or direct proof of BCO prevention; the combined-only design did not test synergy.

## 2. Materials and Methods

### 2.1. Birds, Experimental Design, and Housing

A total of 560 one-day-old male Ross 308 broiler chicks from a single commercial breeder source were used. Birds were inspected on arrival and then vaccinated against Marek’s disease, Newcastle disease, and infectious bronchitis at the hatchery, followed by Gumboro vaccination at day 12 and a Newcastle disease booster at day 14. Chicks were randomly allocated to two dietary treatments with eight replicate pens per treatment and 35 birds per pen: (1) control (Ctrl), a basal diet, and (2) BsP, the basal diet supplemented with *B. subtilis* DSM 29784 (Alterion^®^, Adisseo France S.A.S., Antony, France) at 10 mg/kg feed (10 g per metric tonne), providing 1 × 10^8^ CFU/kg feed, and the commercial phytogenic blend APEX^®^5 (Adisseo France S.A.S., Antony, France) at 150 mg/kg feed (150 g per metric tonne). The phytogenic blend contained a 27.8% garlic fraction; this denotes the garlic ingredient rather than an isolated allicin concentration. Rice bran was used as the replacement ingredient to maintain diet composition. Feed and water were provided ad libitum. Birds received pelleted starter (days 1–21), grower (days 22–35), and finisher (days 36–42) diets formulated with reference to the Ross Broiler Nutrition Specifications (Aviagen, 2022) [25]; ingredient and nutrient compositions are provided in Table 1. All pens were located in a single evaporative-cooled production house. Each pen (1.3 × 1.9 m) was equipped with a feeder and drinker and contained rice-husk litter over the wire-floor system. House temperature and relative humidity were monitored daily. House temperature was approximately 25–28 °C.

The probiotic level of 1 × 10^8^ CFU/kg feed is consistent with previous broiler studies of *B. subtilis* DSM 29784 [14,26]. The phytogenic blend inclusion of 150 g per metric tonne matches the rate described in a published industry report of commercial Ross 308 field use [25,27]. These sources provide prior-use context; the present study evaluated one fixed combination rather than establishing an optimum dose.

### 2.2. Growth Performance

Body weight (BW) was recorded for all birds on days 1, 7, 14, 21, 28, 35, and 42. Feed offered and feed refusals were recorded weekly for each replicate pen to determine feed intake (FI). Body weight gain (BWG) and feed conversion ratio (FCR) were calculated for each feeding phase and for the overall 42-day experimental period. Mortality was monitored and recorded daily, and growth performance data were corrected for mortality before statistical analysis. The pen was the experimental unit for growth performance analysis.

### 2.3. Sample Collection

Birds were observed for lameness every two days from day 15 onward, and each affected bird was counted once. BCO lesions were not systematically assessed. Before scheduled sampling, birds were fasted for 12 h. On days 21, 35, and 42, one bird was randomly selected from the clinically normal birds in each replicate pen and humanely euthanized, yielding eight birds per treatment at each sampling age. Sampled birds showed no visible lameness or other overt clinical abnormalities. Different birds were sampled at successive ages. Jejunal and ileal tissues, cecal digesta, blood, and proximal femoral samples were collected. Intestinal tissues and cecal digesta were placed in sterile tubes, snap-frozen in liquid nitrogen, and stored at −80 °C. Proximal femurs were aseptically excised in a laminar-flow hood using instruments disinfected with 95% ethanol and flame sterilized between samples. The proximal metaphysis and physis were collected, the cut surface was disinfected and flame-sterilized, and the sample was stored at −80 °C. Tissue RNA was used for host gene expression analysis; DNA from cecal digesta and femoral samples was used for microbial profiling and quantitative detection of *Escherichia coli*, *Staphylococcus aureus*, and *Enterococcus cecorum*. Additional femoral material was retained for mineral analysis.

### 2.4. Gene Expression of Gut- and Bone-Related Biomarkers

One bird per pen was analyzed at days 21, 35, and 42 (*n* = 8 per treatment per age). Gene expression was quantified in the jejunum (barrier-associated genes), ileum (inflammatory and calcium phosphate transport-related genes), and femur (inflammatory and osteogenic genes) using real-time quantitative PCR. Primer sequences and annealing temperatures are listed in Table 2. Total RNA was extracted using the RNeasy Mini Kit (Qiagen, Hilden, Germany) according to the manufacturer’s instructions. RNA concentration and purity were assessed with a NanoDrop 2000 spectrophotometer (Thermo Scientific, Waltham, MA, USA), and samples with A260/A280 > 1.8 were retained; RNA integrity was additionally assessed by 1% agarose gel electrophoresis. RNA was diluted to 10 ng/µL and stored at −80 °C until analysis. Reactions were performed on a Bio-Rad CFX instrument using CAPITAL qPCR Green Mix (Biotechrabbit GmbH, Berlin, Germany) in a 20 µL reaction containing 5 µL of 4 × master mix, 0.8 µL of each primer (final concentration of 200 nM), 2 µL of template, and nuclease-free water. Primer-specific cycling conditions were used, followed by melt curve analysis. Samples were analyzed in triplicate. Relative gene expression was normalized to GAPDH and calculated using the 2^−ΔΔCt^ method [28], with results expressed as fold change relative to the calibrator.

### 2.5. 16S rRNA Gene Sequencing and Microbiome Analysis

At days 21, 35, and 42, cecal digesta and proximal femoral samples from one bird per pen were used for microbial community analysis. Samples were preserved with DNA/RNA Shield (R1100; Zymo Research, Irvine, CA, USA). Genomic DNA was extracted using the DNeasy PowerSoil Pro Kit (Qiagen, Hilden, Germany) according to the manufacturer’s instructions. DNA quantity was assessed before amplification of the V6-V8 region of the bacterial 16S rRNA gene. The V6–V8 region was amplified using the primers reported in the original protocol (forward: 5′-TCGTCGGCAGCGTCAGATGTGTATAAGAGACAGAA TTGACGGGGCCCGC-3′; reverse: 5′-GTCTCGTGGGCTCGGAGATGTGTATAAGACAGACGGGC GTGWGTCAA-3′). PCRs used Phusion Hot Start II High-Fidelity PCR Master Mix (Thermo Fisher Scientific, Waltham, MA, USA), 0.2 µM of each primer, and approximately 10 ng of template DNA. Cycling consisted of 95 °C for 3 min; 25 cycles of 95 °C for 30 s, 55 °C for 30 s, and 72 °C for 30 s; and a final extension at 72 °C for 5 min. Amplicons (~550 bp) were verified by 1.5% agarose gel electrophoresis, quantified using a QFX Fluorometer (DeNovix, Wilmington, DE, USA), assessed with a QIAxcel Advanced system (Qiagen), purified using AMPure XP beads (Beckman Coulter, Indianapolis, IN, USA), and indexed with the Nextera XT Index Kit. Indexed amplicons were pooled and sequenced using paired-end chemistry (2 × 250 bp) on an Illumina MiSeq platform at Prince of Songkla University, Songkhla, Thailand. Sequence processing used QIIME 1.9.1 with uclust-based open-reference OTU picking at 97% similarity against the PKSS 4.0 reference database, as reported in the original analytical workflow. Alpha diversity was summarized using Chao1 and Shannon indices. Beta diversity was assessed using Bray–Curtis dissimilarity and visualized by principal coordinate analysis (PCoA). Predicted microbial functional potential was inferred from the cecal 16S rRNA gene data using PICRUSt2 [29]. The BIOM format OTU table was processed with a Nearest Sequenced Taxon Index (NSTI) cutoff of 2.0 using q2-picrust2 in QIIME2 (version 2019.10). Predicted gene content was annotated to KEGG Orthologs and Enzyme Commission numbers, internally normalized, and mapped to MetaCyc pathways [30,31]. Because these functions were inferred from 16S rRNA gene profiles rather than directly measured, they are interpreted as predicted metabolic potential.

### 2.6. Blood Hormones and Mineral Analyses

Blood was collected on days 21, 35, and 42 into heparinized tubes and centrifuged at 825× *g* for 15 min at 4 °C. Corticosterone and serotonin were quantified by HPLC as described previously [32]. Briefly, 1 mL of sample was combined with an internal standard (dexamethasone) and 0.6 mL of sodium hydroxide, extracted twice with dichloromethane, pooled, evaporated under nitrogen at 40 °C, reconstituted in methanol:water (60:40, *v*/*v*), and filtered through a 0.45 µm nylon membrane. Separation was performed using an Alliance e2695 HPLC system with a Waters 2998 photodiode array detector (Waters, Milford, MA, USA), a Purospher STAR RP-18 endcapped column (Merck, Darmstadt, Germany), and isocratic methanol:water (60:40, *v*/*v*) at 1.0 mL/min; the injection volume was 20 µL. Quantification was based on analyte-to-internal-standard peak-area ratios. Serum phosphate was determined colorimetrically using molybdate and hydroquinone-ascorbate reagents, with absorbance measured at 578 nm. Serum calcium was measured using an o-cresolphthalein complexone method with absorbance at 565 nm [33]. For femoral mineral analysis, bone samples were ashed at ≤550 °C and acid-digested. Calcium was quantified using the o-cresolphthalein complexone method, and phosphorus was measured using the molybdate-based colorimetric procedure according to the analytical protocol described in the original study [33,34]. The results were expressed relative to bone sample mass.

### 2.7. Quantitative Detection of BCO-Associated Bacteria in Cecal and Femoral Samples

*Escherichia coli*, *Enterococcus cecorum*, and *Staphylococcus aureus* were quantified in cecal digesta and femoral samples by digital PCR using the QIAcuity platform. DNA was extracted using the DNeasy PowerSoil Pro Kit (Qiagen, Hilden, Germany), quantified with a QIAxpert spectrophotometer (Qiagen, Hilden, Germany), and adjusted to 10 ng/µL before analysis. Reference DNA from quantified bacterial cultures was used during assay verification. Primer sequences and annealing temperatures are listed in Table 2. Target-specific primers were designed using NCBI Primer-BLAST [35]. Serial dilutions of reference DNA were used to assess assay performance across a defined concentration range. Each 12 µL digital PCR reaction contained 4 µL of 3 × EvaGreen PCR Master Mix (Qiagen, Hilden, Germany), forward and reverse primers at a final concentration of 0.4 µM each, 1 µL of template DNA, and nuclease-free water. Reactions were loaded into QIAcuity 24-well nanoplates and analyzed on a QIAcuity One instrument (Qiagen, Hilden, Germany). Sensitivity, specificity, and inhibition were assessed during assay verification. Data were analyzed using QIAcuity Software Suite version 2.5.0.1. (Qiagen, Hilden, Germany). Thresholds separating positive and negative partitions were defined relative to negative controls, and reactions with >1000 accepted partitions were retained. Concentrations were reported by the instrument software.

### 2.8. Age-Specific Microbiome–Gut–Bone Network Analysis

Age-specific correlation network analysis was used to characterize associations among cecal microbial features, intestinal health markers, and femoral inflammatory and osteogenic responses at days 21, 35, and 42. Analyses were conducted separately by age. Pairwise associations were calculated using Spearman’s rank correlation coefficient (rho). Microbial features comprised selected bacterial taxa and the Shannon diversity index. Host variables comprised jejunal barrier-associated markers (*CLDN-1*, *OCLD-1*, *TJP-1*, and *MUC-2*), ileal inflammatory and mineral-transport-related markers (*TNF- α*, *NF-κB*, *cCaSR*, and *NaPi-IIb*), and femoral inflammatory and osteogenic markers (*IL-6*, *IL-17*, *TNF-α*, *NLRP3*, *BMP-2*, *SMAD-1*, *SPARC*, and *RUNX2*). The original day-21 and day-35 analyses retained associations with |ρ| ≥ 0.50 and *p* < 0.05. The day-42 network was reconstructed from the supplied retained-edge export (|ρ| ≥ 0.50); this export contains correlation coefficients but no *p*-values, so its significance filtering could not be independently checked. Networks were represented as undirected graphs; node classes corresponded to microbiome, gut health, and bone-associated domains, and edge width was scaled to |rho|. Network topology was summarized by node and edge counts, density, correlation-sign distribution, and cross-domain edge counts. Visualization was performed in Python version 3.13.9 using NetworkX version 3.6.1and Matplotlib version 3.10.9 with age-specific displays. The day-42 display uses the same spatial and color scheme as panel B, with microbial features on the left, intestinal markers on the right, and femoral markers above. All exported nodes and edges were retained; selected taxon labels are shown to reduce overlap, and a full source-to-display key is supplied. Network edges were interpreted as statistical associations rather than directional or causal relationships.

### 2.9. Statistical Analysis

Statistical analyses were conducted using GraphPad Prism version 10.4.0 (GraphPad Software, La Jolla, CA, USA), R version 4.4.2, and the microbiome analysis tools described above. Growth performance variables were compared between dietary treatments using Student’s *t*-test with pen as the experimental unit. For host biomarkers, hormones, minerals, and targeted bacterial abundance, between-treatment comparisons were performed at each sampling age using the multiple *t*-test procedure reported in the original analysis. For microbiome data, alpha diversity (Chao1 and Shannon) was compared using the Kruskal–Wallis test. Bray–Curtis dissimilarities were visualized by PCoA and assessed by PERMANOVA (adonis). Differential taxonomic comparisons used Kruskal–Wallis tests with Benjamini correction at a 1% false-discovery rate, while DESeq2 was additionally used for OTU-level differential abundance analysis. Predicted MetaCyc pathway abundances were compared in STAMP version 2.1.3 using the Kruskal–Wallis test. Unless otherwise specified, significance was defined as *p* < 0.05. Network filtering is described in Section 2.8. Clinical lameness counts were summarized descriptively without an inferential between-treatment comparison.

## 3. Results

### 3.1. Growth Performance

Growth performance is summarized in Table 3. Initial BW did not differ between treatments. BsP reduced FCR during days 8–14, 29–35, and 36–42 and across the overall 1–42-day period (*p* < 0.05). During days 36–42, birds receiving BsP also had higher BW and BWG than the controls (*p* < 0.05). Consequently, the final BW and cumulative BWG over days 1–42 were greater in the BsP group, whereas cumulative feed intake and mortality did not differ significantly between treatments.

### 3.2. Intestinal Barrier, Inflammation, and Calcium Phosphate Transport-Related Gene Expression

**Figure 1:**
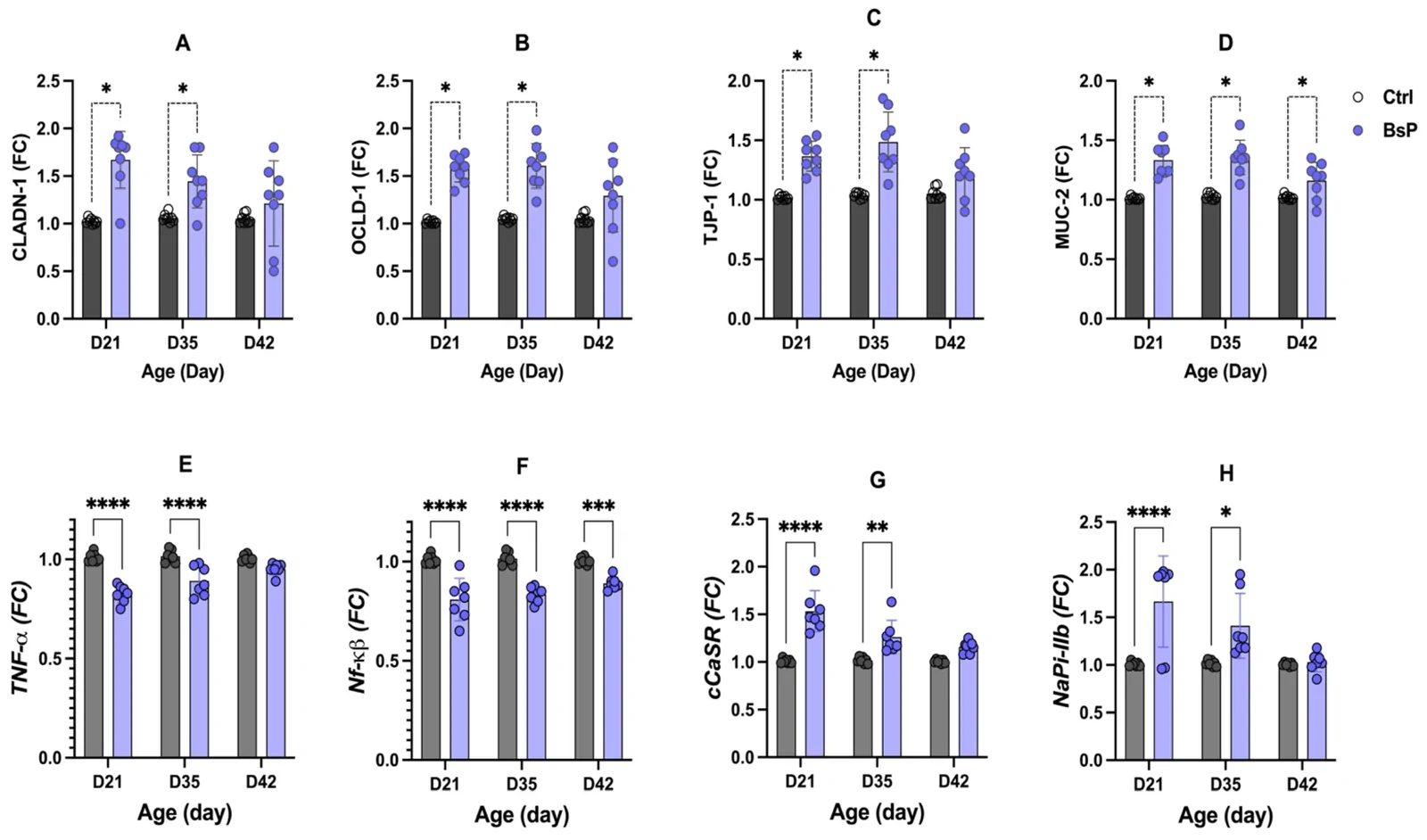
Relative gene expression (fold change) of jejunal barrier markers: claudin-1 (*CLDN-1*; (**A**)), occludin-1 (*OCLD-1*; (**B**)), tight junction protein-1 (*TJP-1*; (**C**)), and mucin-2 (*MUC-2*; (**D**)); ileal inflammatory markers tumor necrosis factor alpha (*TNF-α*; (**E**)) and nuclear factor-kappaB (*NF-κB*; (**F**)); and ileal calcium phosphate transport-related markers calcium-sensing receptor (*cCaSR*; (**G**)) and sodium phosphate cotransporter IIb (*NaPi-IIb*; (**H**)) at days 21, 35, and 42. Broilers received the basal control diet (Ctrl) or *Bacillus subtilis* DSM 29784 combined with a garlic-based phytogenic blend (BsP). * *p* < 0.05, ** *p* < 0.01, *** *p* < 0.001, and **** *p* < 0.0001.

Jejunal expression of *CLDN-1* and *OCLD-1* was higher in BsP-supplemented birds at days 21 and 35, *TJP-1* was higher at days 21 and 35, and *MUC-2* was higher at days 21, 35, and 42 (*p* < 0.05; Figure 1A–D). In the ileum, *TNF-α* expression was lower at days 21 and 35, and *NF-κB* expression was lower at days 21, 35, and 42 in BsP-fed birds (*p* < 0.05; Figure 1E,F). Expression of *cCaSR* and *NaPi-IIb* was higher at days 21 and 35 (*p* < 0.05), whereas differences at day 42 were not significant (Figure 1G,H).

### 3.3. Femoral Inflammatory and Osteogenic Gene Expression

**Figure 2:**
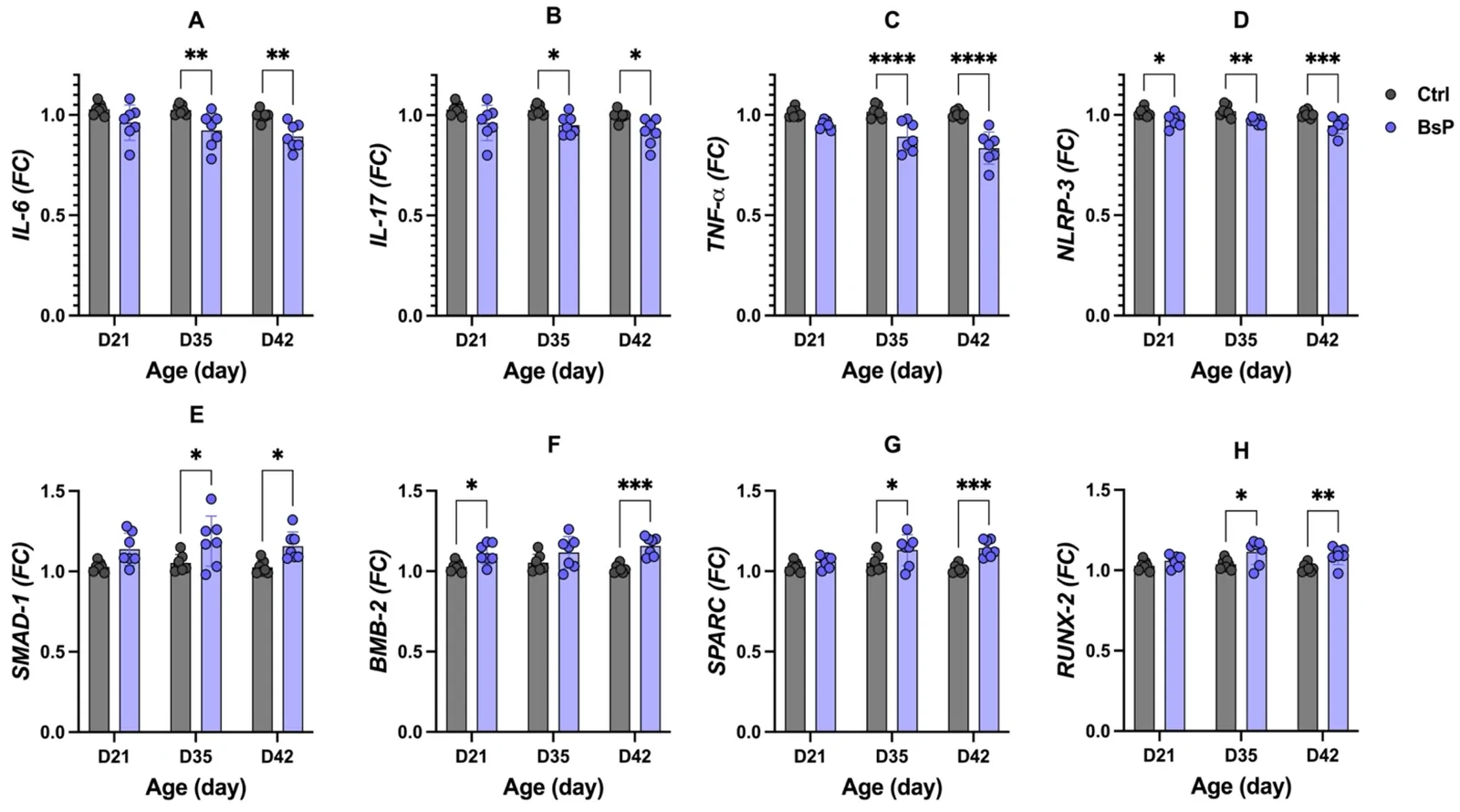
Relative femoral gene expression (fold change) of inflammatory markers interleukin-6 (*IL-6*; (**A**)), interleukin-17 (*IL-17*; (**B**)), tumor necrosis factor alpha (*TNF-α*; (**C**)), and NLR family pyrin domain-containing 3 (*NLRP3*; (**D**)), and osteogenic markers bone morphogenetic protein-2 (*BMP-2*; (**E**)), osteonectin (*SPARC*; (**F**)), SMAD family member 1 (*SMAD-1*; (**G**)), and runt-related transcription factor 2 (*RUNX2*; (**H**)) at days 21, 35, and 42. Broilers received the basal control diet (Ctrl) or *Bacillus subtilis* DSM 29784 combined with a garlic-based phytogenic blend (BsP). Between-treatment differences at each age were analyzed using the multiple *t*-test procedure described; * *p* < 0.05, ** *p* < 0.01, *** *p* < 0.001, and **** *p* < 0.0001.

Femoral gene expression responses are shown in Figure 2. BsP reduced NLRP3 expression at days 21, 35, and 42 and reduced *IL-6*, *IL-17*, and *TNF-α* at selected ages, with the clearest reductions at days 35 and 42 (*p* < 0.05). In contrast, osteogenic markers were increased at selected time points: BMP-2 was higher at days 21 and 42, whereas *SMAD-1*, *SPARC*, and *RUNX2* were higher at days 35 and 42 (*p* < 0.05).

### 3.4. Blood Hormones and Mineral Status

Hormone and mineral data are presented in Table 4. Serotonin concentrations were higher and corticosterone concentrations were lower in BsP-fed birds at days 21, 35, and 42 (*p* < 0.05). Serum calcium was higher at days 21 and 35, and serum phosphate was higher at days 21 and 35, with a borderline difference at day 42 (*p* = 0.05). Femoral calcium and phosphate concentrations were higher in the BsP group at days 21 and 35 (*p* < 0.05), whereas no significant treatment differences were observed at day 42.

### 3.5. Cecal and Femoral Microbial Community Profiles

**Figure 3:**
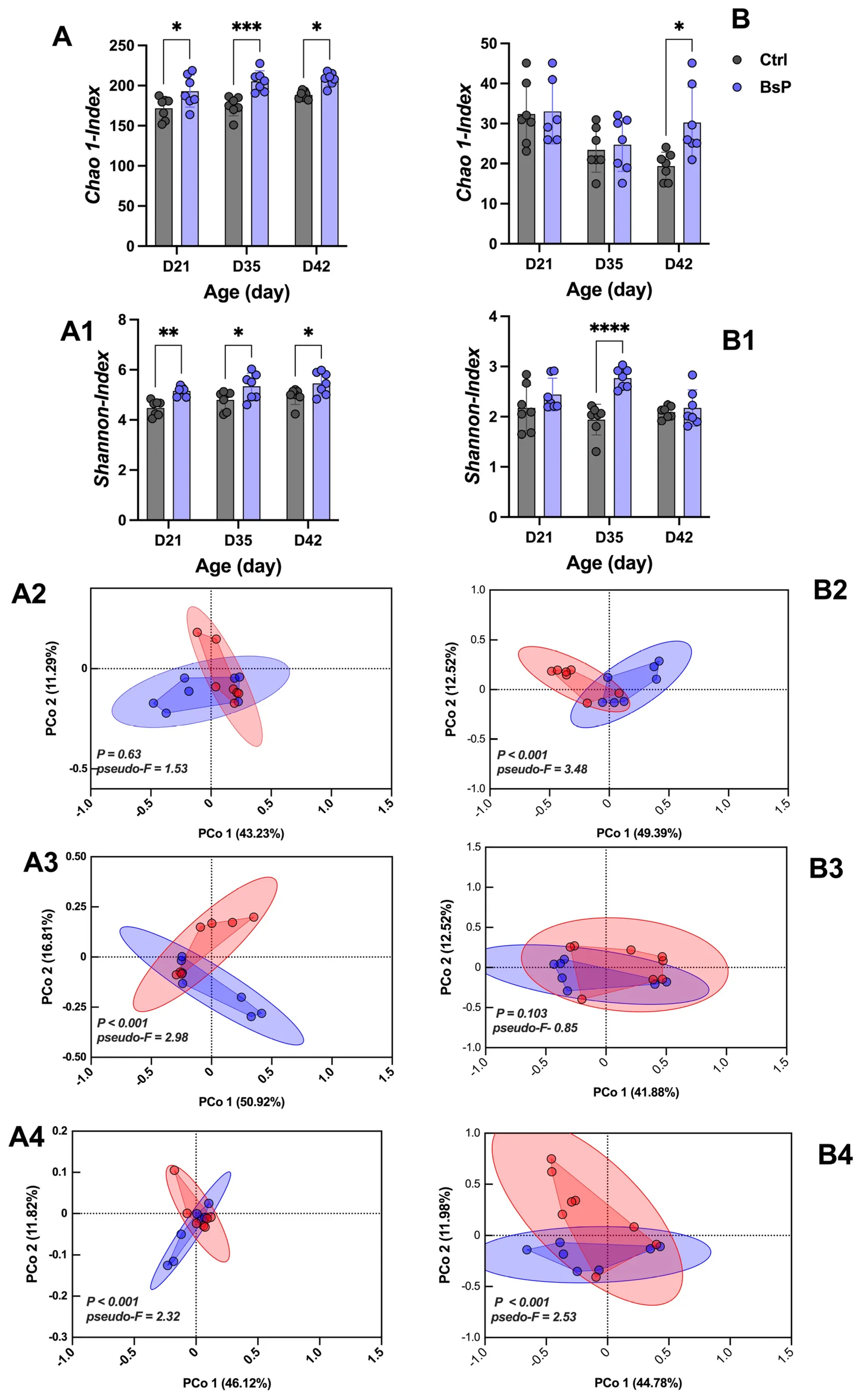
Alpha and beta diversity of cecal and femoral bacterial DNA profiles. Chao1 richness and Shannon diversity are shown for cecal (**A**,**A1**) and femoral (**B**,**B1**) samples. Bray–Curtis principal coordinate analysis (PCoA) is shown for cecal samples at days 21 (**A2**), 35 (**A3**), and 42 (**A4**), and femoral samples at days 21 (**B2**), 35 (**B3**), and 42 (**B4**). Broilers received the basal control diet (Ctrl) or *Bacillus subtilis* DSM 29784 combined with a garlic-based phytogenic blend (BsP). Alpha-diversity comparisons used the Kruskal–Wallis test; * *p* < 0.05, ** *p* < 0.01, *** *p* < 0.001, and **** *p* < 0.0001. Beta-diversity differences were assessed by PERMANOVA on Bray–Curtis dissimilarities; *p* < 0.001 where indicated. Each point represents an individual sample.

**Figure 4:**
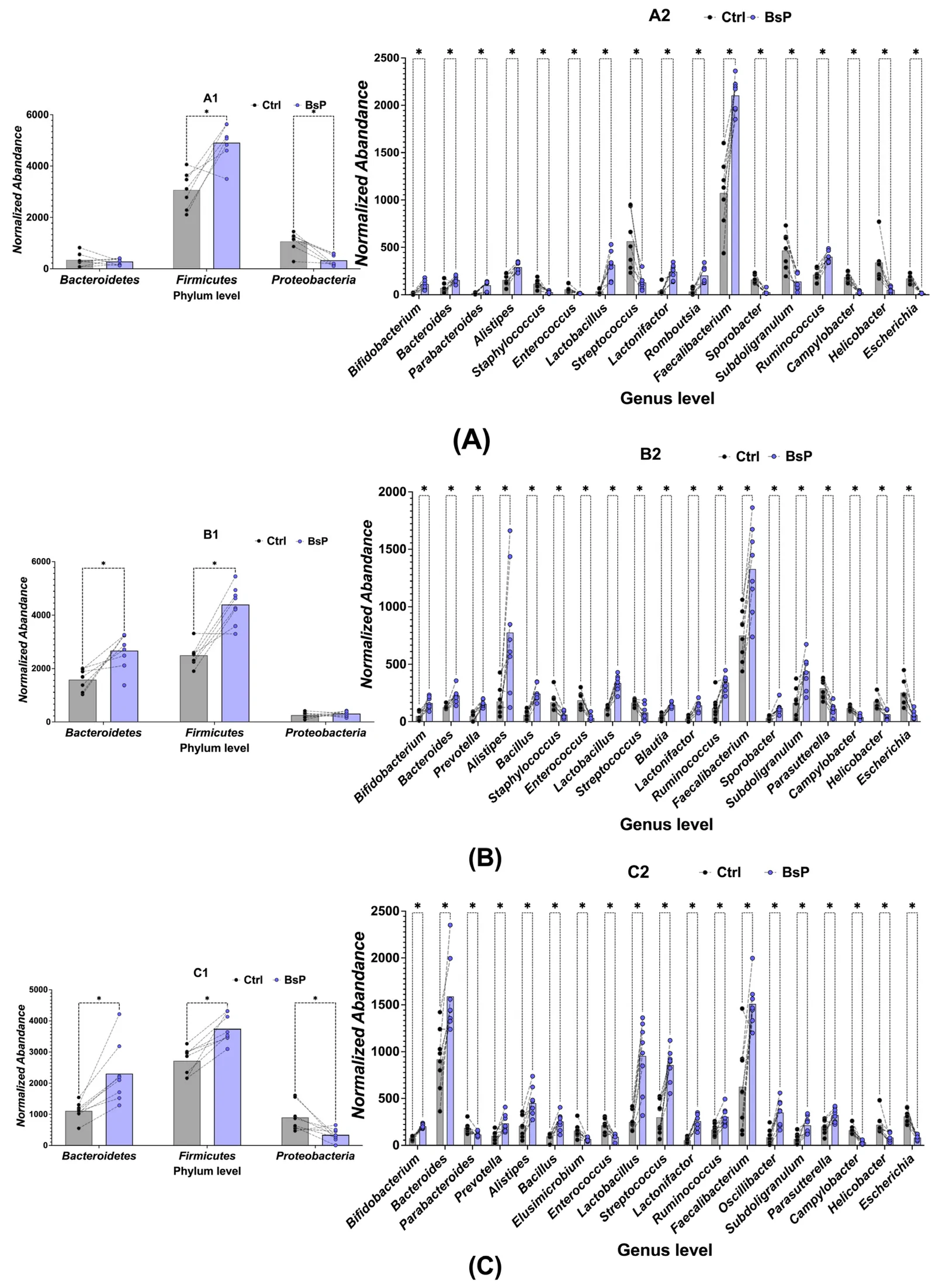
Relative abundance of major phyla and treatment-associated phylum and genera in the cecal bacterial community at days 21 (**A**,**A1**,**A2**), 35 (**B**,**B1**,**B2**), and 42 (**C**,**C1**,**C2**). Broilers received the basal control diet (Ctrl) or *Bacillus subtilis* DSM 29784 combined with a garlic-based phytogenic blend (BsP). Differential comparisons used the Kruskal–Wallis test with Benjamini correction at a 1% false-discovery rate; * *p* < 0.05.

**Figure 5:**
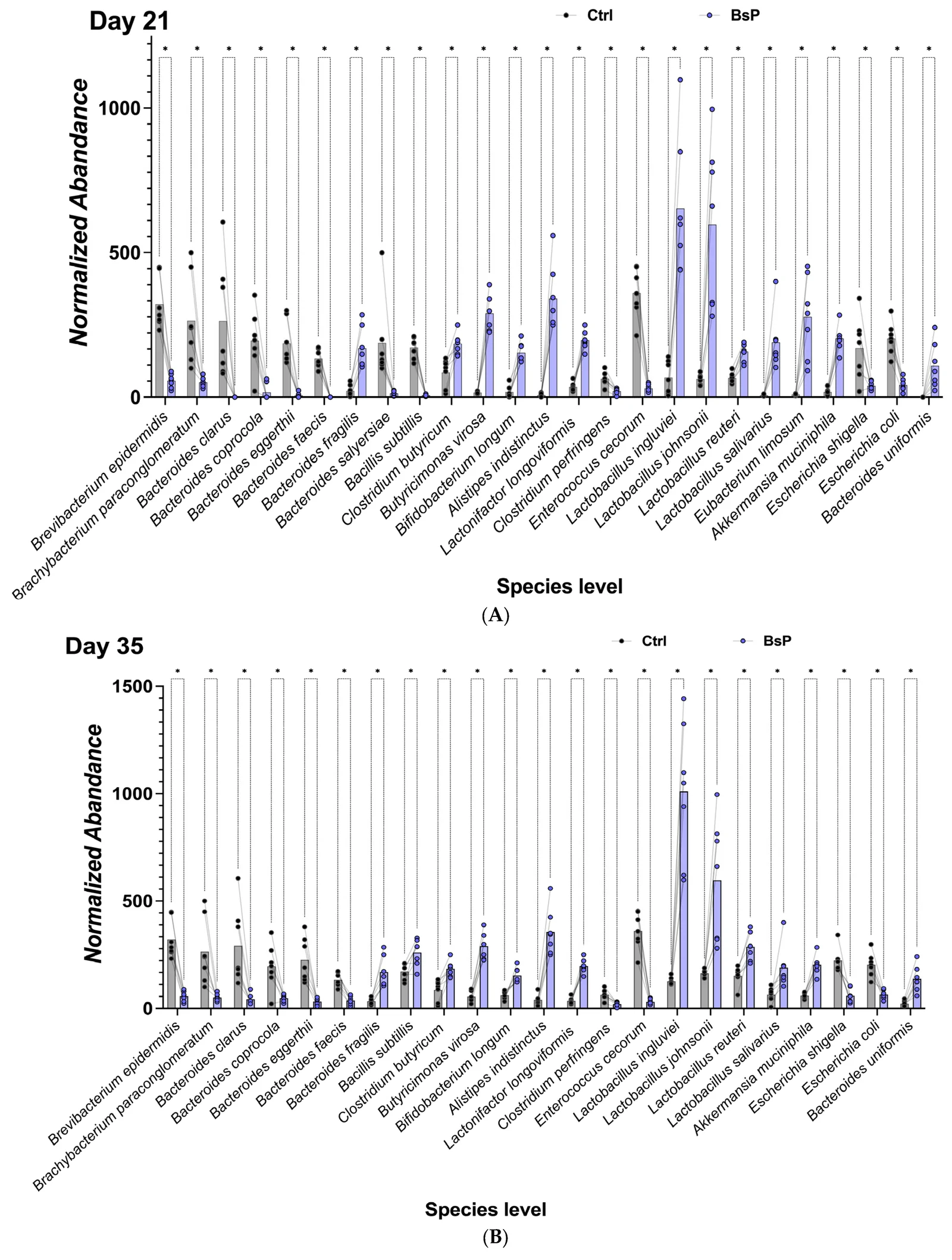

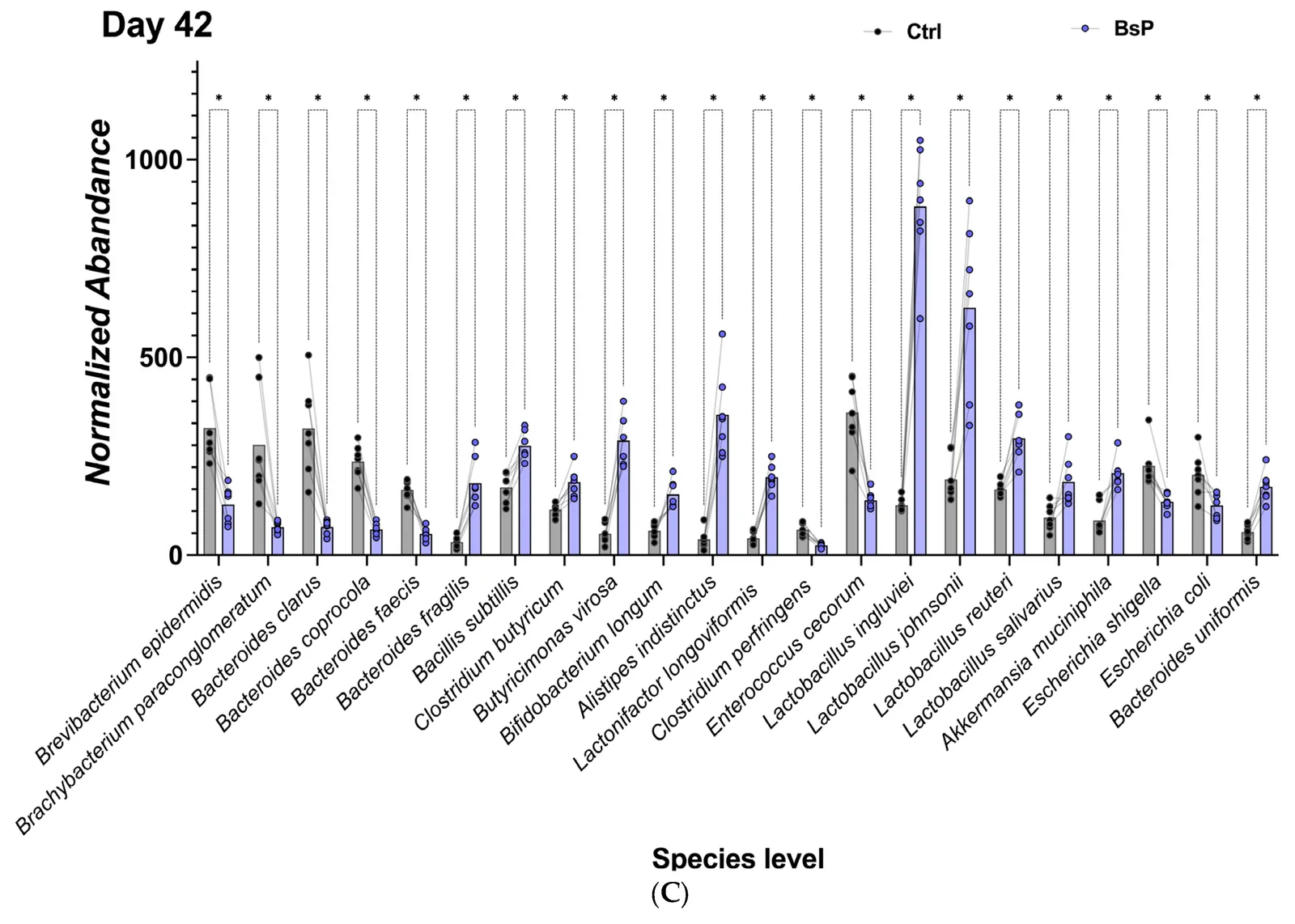
(**A**) Relative abundance of major species in the cecal microbiome at day 21. Broilers received the basal control diet (Ctrl) or *Bacillus subtilis* DSM 29784 combined with a garlic-based phytogenic blend (BsP). Differential comparisons used the Kruskal–Wallis test with Benjamini correction at a 1% false-discovery rate; * *p* < 0.05. (**B**) Relative abundance of major species in the cecal microbiome at day 35. Broilers received the basal control diet (Ctrl) or *Bacillus subtilis* DSM 29784 combined with a garlic-based phytogenic blend (BsP). Differential comparisons used the Kruskal–Wallis test with Benjamini correction at a 1% false-discovery rate; * *p* < 0.05. (**C**) Relative abundance of major species in the cecal microbiome at day 42. Broilers received the basal control diet (Ctrl) or *Bacillus subtilis* DSM 29784 combined with a garlic-based phytogenic blend (BsP). Differential comparisons used the Kruskal–Wallis test with Benjamini correction at a 1% false-discovery rate; * *p* < 0.05.

A total of 5,049,408 raw reads were generated. After assembly and quality control, 1,519,108 sequences were retained, averaging 52,598 reads per sample with a mean read length of 420 bp. Good’s coverage exceeded 99%. In the cecum, BsP increased Chao1 richness and Shannon diversity at days 21, 35, and 42 (*p* < 0.05; Figure 3A,A1). Bray–Curtis PCoA demonstrated treatment-related separation of cecal communities at the sampled ages (PERMANOVA, *p* < 0.001; Figure 3A2–A4). Firmicutes, Bacteroidetes, and Proteobacteria dominated the cecal community. BsP was associated with higher Firmicutes abundance across the sampled ages, higher Bacteroidetes at days 35 and 42, and lower Proteobacteria at days 21 and 42 (*p* < 0.05; Figure 4). At the genus and species levels, treatment-associated shifts included increases in several taxa commonly associated with intestinal fermentation or probiotic activity and decreases in several potentially pathogenic taxa (Figure 4 and Figure 5). Species reported as increased included *Clostridium butyricum*, *Enterococcus faecium*, *Lactobacillus salivarius*, *L. crispatus*, and *Bifidobacterium longum*, whereas *Escherichia coli*, *Clostridium perfringens*, and *Enterococcus cecorum* were among taxa reported as decreased (*p* < 0.05).

**Figure 6:**
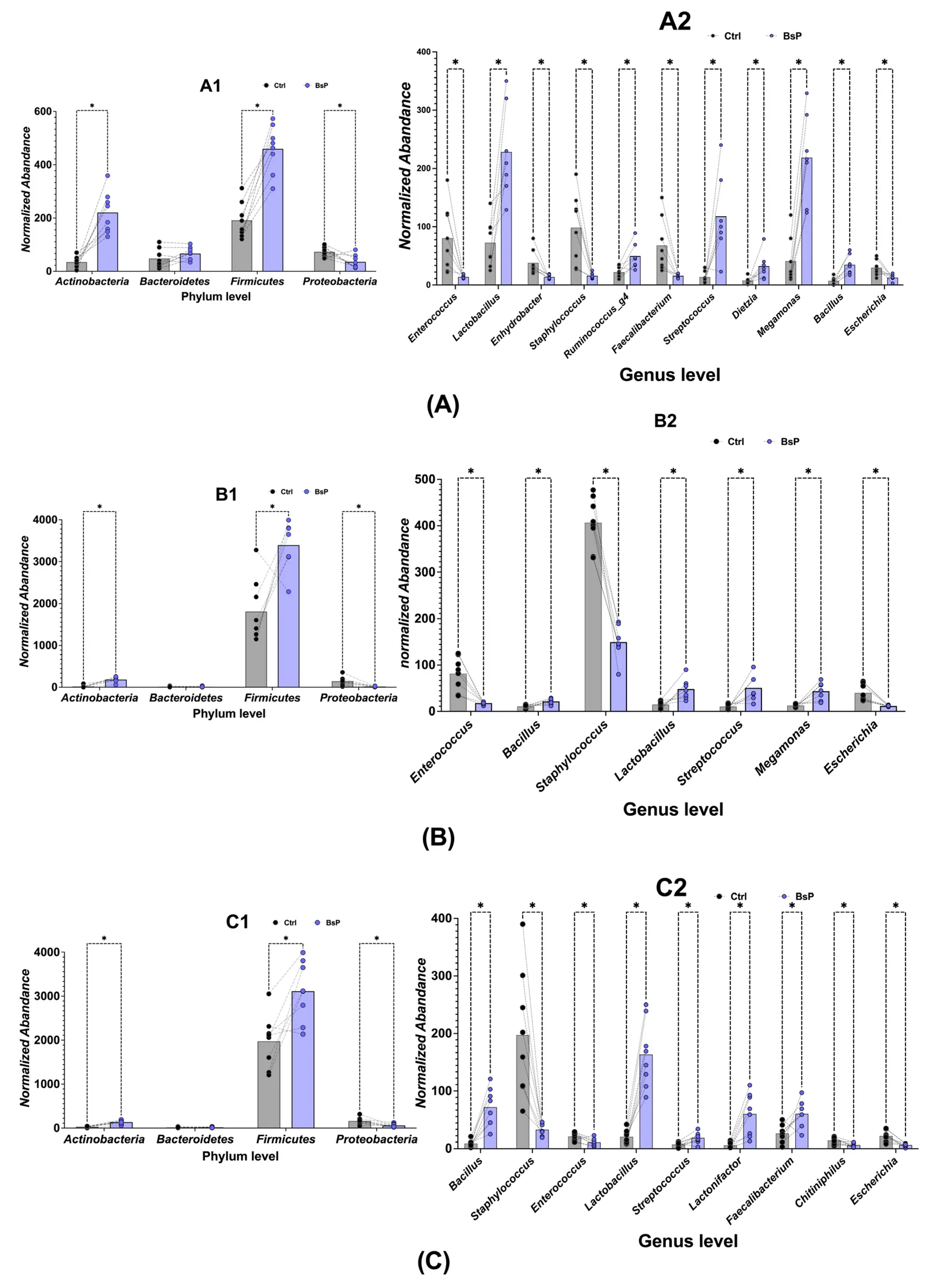
(**A**) Relative abundance of major phylum (**A1**) and species (**A2**) in the femoral microbiome at day 21. Broilers received the basal control diet (Ctrl) or *Bacillus subtilis* DSM 29784 combined with a garlic-based phytogenic blend (BsP). Differential comparisons used the Kruskal–Wallis test with Benjamini correction at a 1% false-discovery rate; * *p* < 0.05. (**B**) Relative abundance of major phylum (**B1**) and species (**B2**) in the femoral microbiome at day 35. Broilers received the basal control diet (Ctrl) or *Bacillus subtilis* DSM 29784 combined with a garlic-based phytogenic blend (BsP). Differential comparisons used the Kruskal–Wallis test with Benjamini correction at a 1% false-discovery rate; * *p* < 0.05. (**C**) Relative abundance of major phylum (**C1**) and species (**C2**) in the femoral microbiome at day 42. Broilers received the basal control diet (Ctrl) or *Bacillus subtilis* DSM 29784 combined with a garlic-based phytogenic blend (BsP). Differential comparisons used the Kruskal–Wallis test with Benjamini correction at a 1% false-discovery rate; * *p* < 0.05.

Femoral samples showed a smaller but detectable diversity response. Shannon diversity was higher at day 35 and Chao1 richness was higher at day 42 in BsP-fed birds (*p* < 0.05; Figure 3B,B1). Bray–Curtis PCoA also showed treatment-related differences in femoral microbial profiles at selected ages (PERMANOVA, *p* < 0.001; Figure 3B2–B4). Firmicutes, Bacteroidetes, Proteobacteria, and Actinobacteria were the dominant phyla detected in femoral samples (Figure 6). Because femoral tissue is a low-biomass matrix, these 16S rRNA gene-based profiles should be interpreted as bacterial DNA signatures rather than direct evidence of viable bone colonization.

### 3.6. Predicted Cecal Microbial Functional Potential

**Figure 7:**
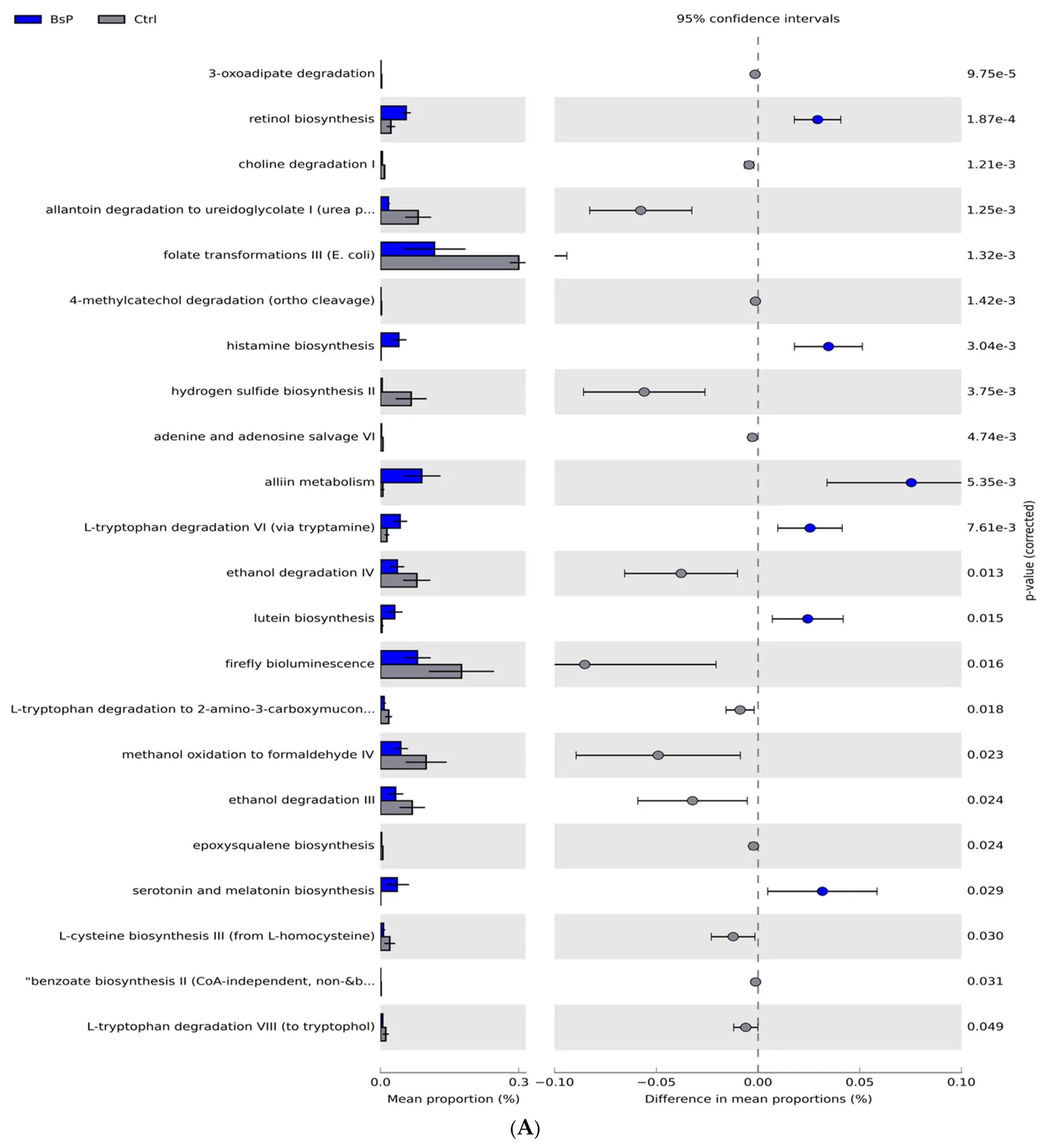

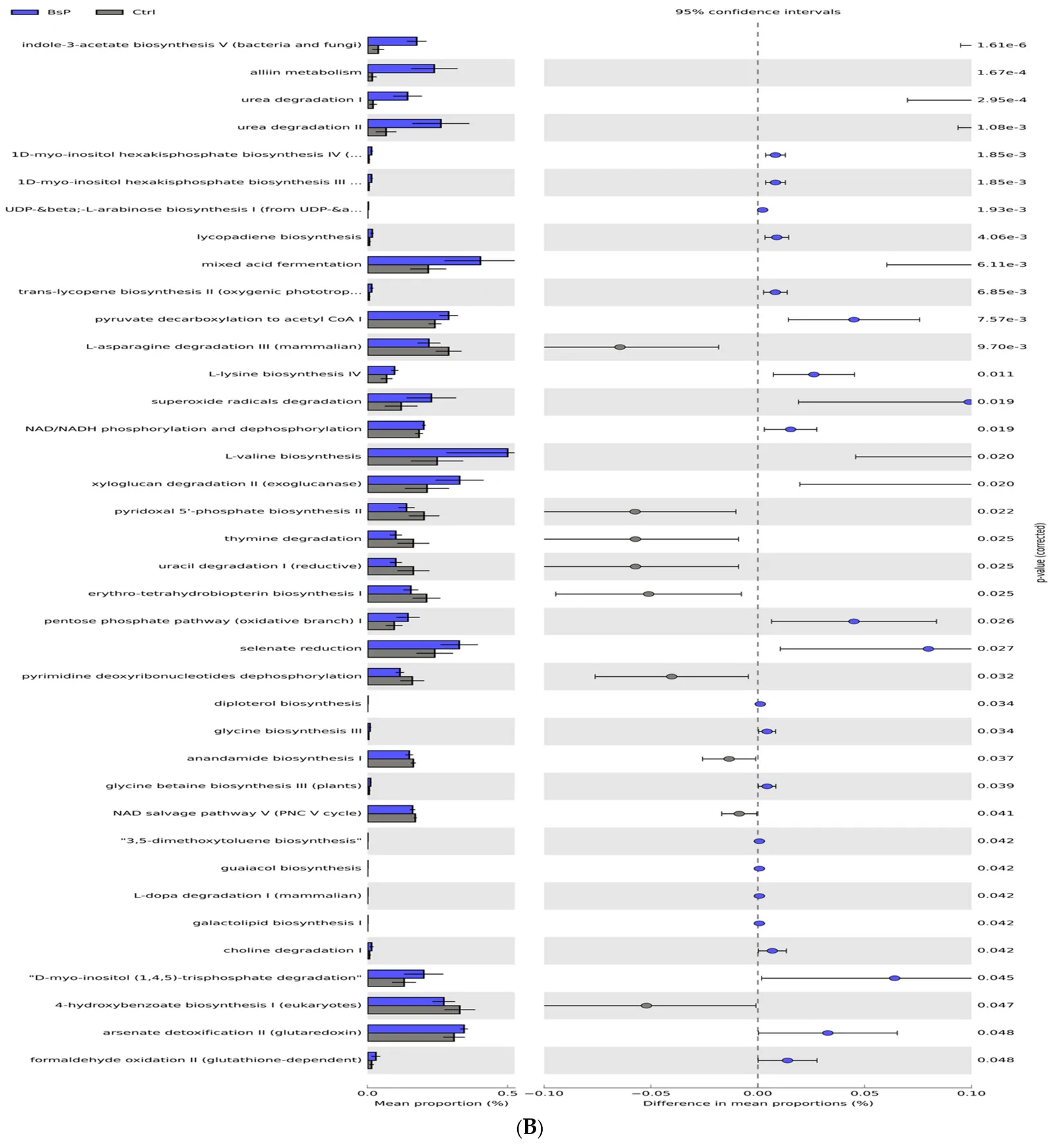

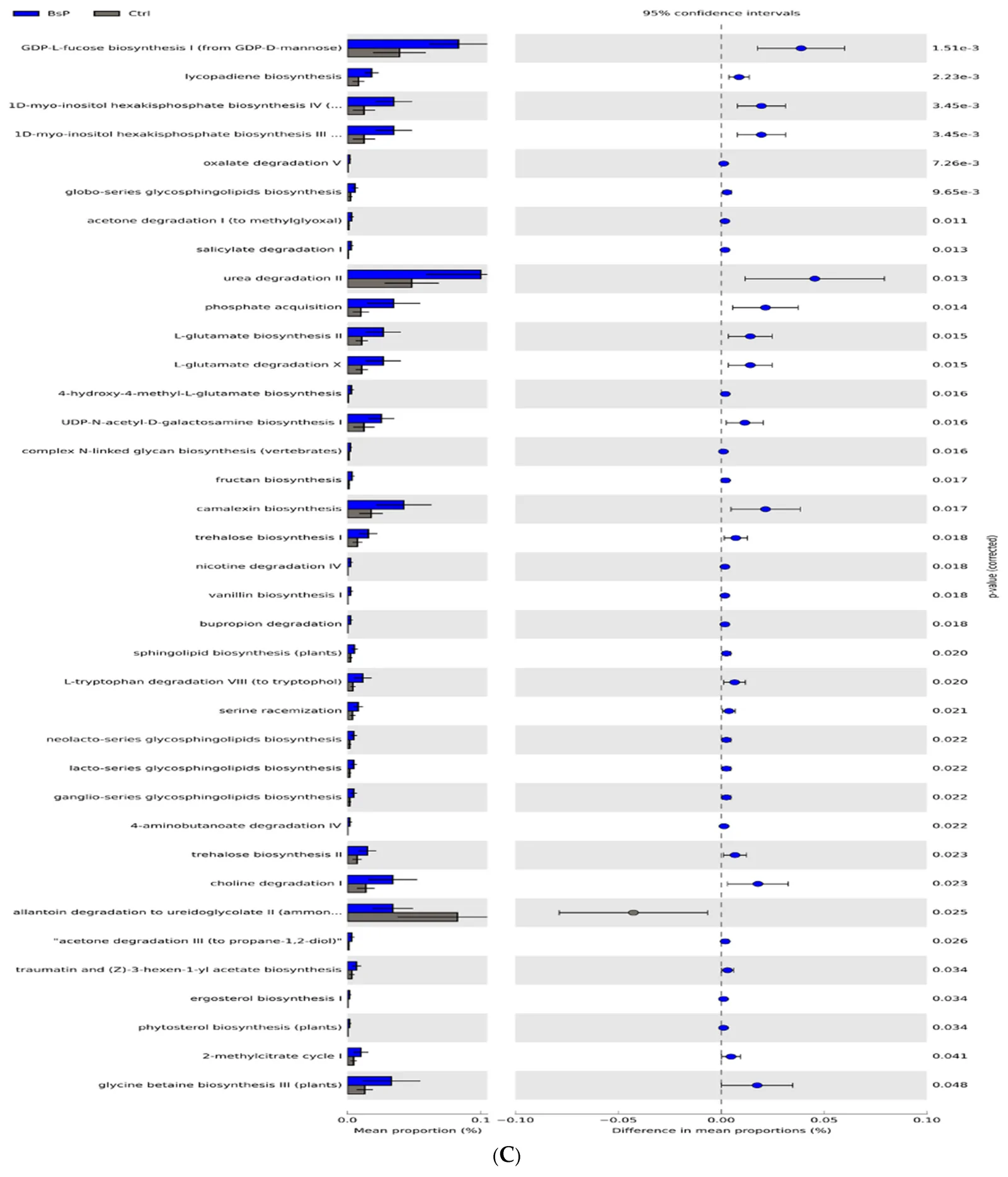
(**A**) Significant MetaCyc pathways in cecal microbiome at day 21. Broilers received the basal control diet (Ctrl) or *Bacillus subtilis* DSM 29784 combined with a garlic-based phytogenic blend (BsP). Differential comparisons used the Kruskal–Wallis test with Benjamini correction at a 1% false-discovery rate; *p* < 0.05. The left side of the figure displays the mean pathway proportions (%); the right side illustrates the differences in mean proportions with 95% confidence intervals. (**B**) Significant MetaCyc pathways in cecal microbiome at day 35. Broilers received the basal control diet (Ctrl) or *Bacillus subtilis* DSM 29784 combined with a garlic-based phytogenic blend (BsP). Differential comparisons used the Kruskal–Wallis test with Benjamini correction at a 1% false-discovery rate; *p* < 0.05. The left side of the figure displays the mean pathway proportions (%); the right side illustrates the differences in mean proportions with 95% confidence intervals. (**C**) Significant MetaCyc pathways in cecal microbiome at day 42. Broilers received the basal control diet (Ctrl) or *Bacillus subtilis* DSM 29784 combined with a garlic-based phytogenic blend (BsP). Differential comparisons used the Kruskal–Wallis test with Benjamini correction at a 1% false-discovery rate; *p* < 0.05. The left side of the figure displays the mean pathway proportions (%); the right side illustrates the differences in mean proportions with 95% confidence intervals.

PICRUSt2 analysis identified treatment-associated differences in predicted MetaCyc pathway abundance (Figure 7A–C). At day 21, BsP was associated with higher predicted abundance of pathways related to retinol biosynthesis, histamine and alliin metabolism, L-tryptophan metabolism, and serotonin and melatonin biosynthesis, whereas predicted allantoin degradation and hydrogen-sulfide-related functions were lower. At day 35, treatment-associated differences included pathways related to tocopherol, plastoquinol-9, phosphate acquisition, L-glutamate metabolism, choline metabolism, and L-tryptophan metabolism. At day 42, the supplied STAMP output showed higher predicted representation of phosphate acquisition, L-glutamate biosynthesis II, L-glutamate degradation X, L-tryptophan degradation VIII (to tryptophol), and choline degradation I in BsP, whereas allantoin degradation to ureidoglycolate II was lower (Figure 7C; displays the corrected *p*-values < 0.05). These findings describe inferred pathway representation rather than measured metabolite production or pathway activity.

### 3.7. BCO-Associated Bacteria in Cecal and Femoral Samples

**Figure 8:**
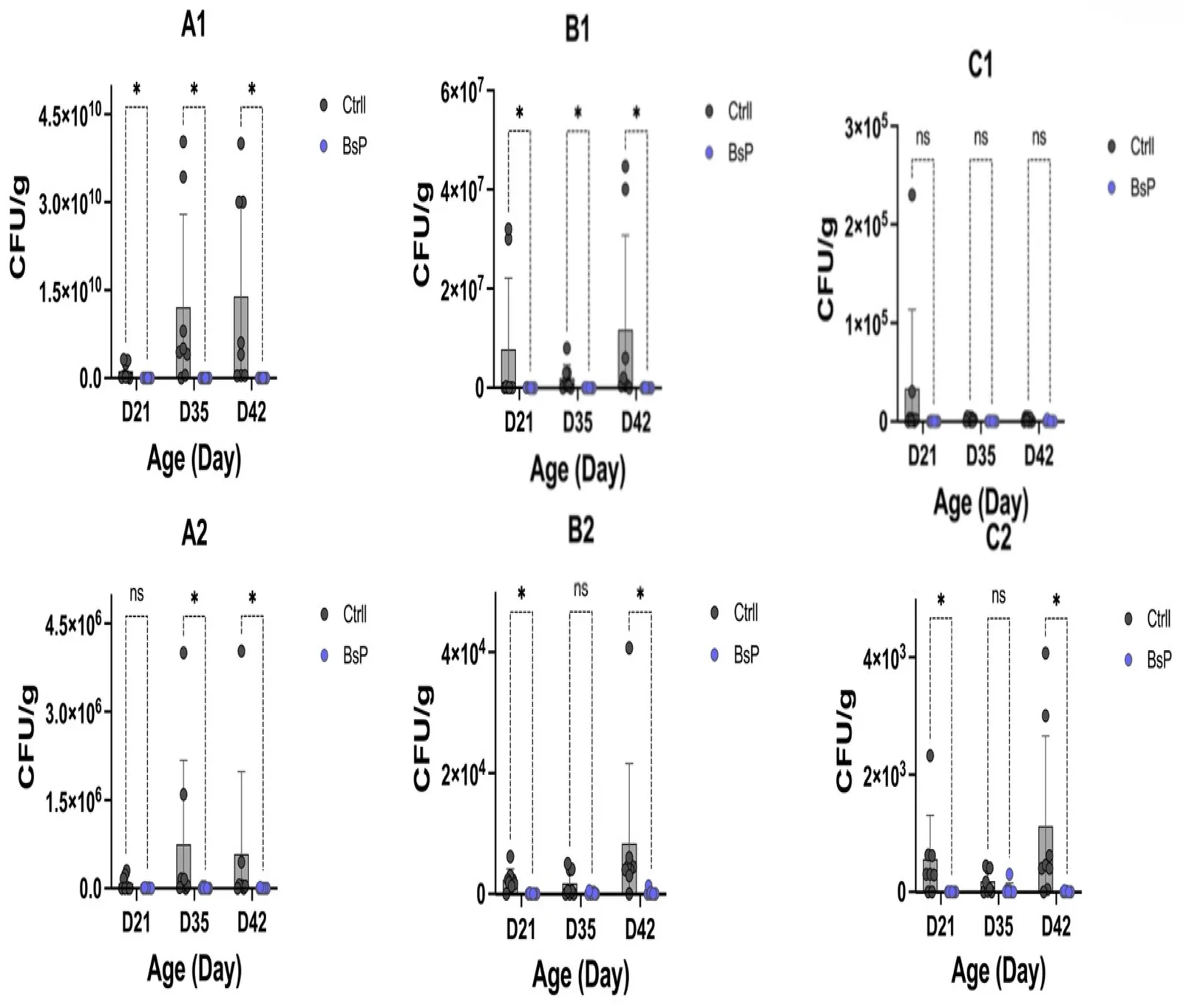
Digital PCR (dPCR) quantification of *Escherichia coli* (**A1**,**A2**), *Enterococcus cecorum* (**B1**,**B2**), and *Staphylococcus aureus* (**C1**,**C2**) in cecal and femoral samples at days 21, 35, and 42. Broilers received the basal control diet (Ctrl) or *Bacillus subtilis* DSM 29784 combined with a garlic-based phytogenic blend (BsP). Between-treatment differences at each age were analyzed using the multiple *t*-test; * *p* < 0.05; ns means non significant.

Digital PCR showed that BsP reduced *E. coli* abundance in cecal samples at all three ages and in femoral samples at days 35 and 42 (*p* < 0.05; Figure 8A1,A2). *E. cecorum* was lower in cecal samples at all three ages and in femoral samples at days 21 and 42 (*p* < 0.05; Figure 8B1,B2). *S. aureus* was lower in femoral samples at days 21 and 42 (*p* < 0.05; Figure 8C1,C2).

### 3.8. Age-Dependent Microbiome–Gut–Bone Interaction Networks

**Figure 9:**
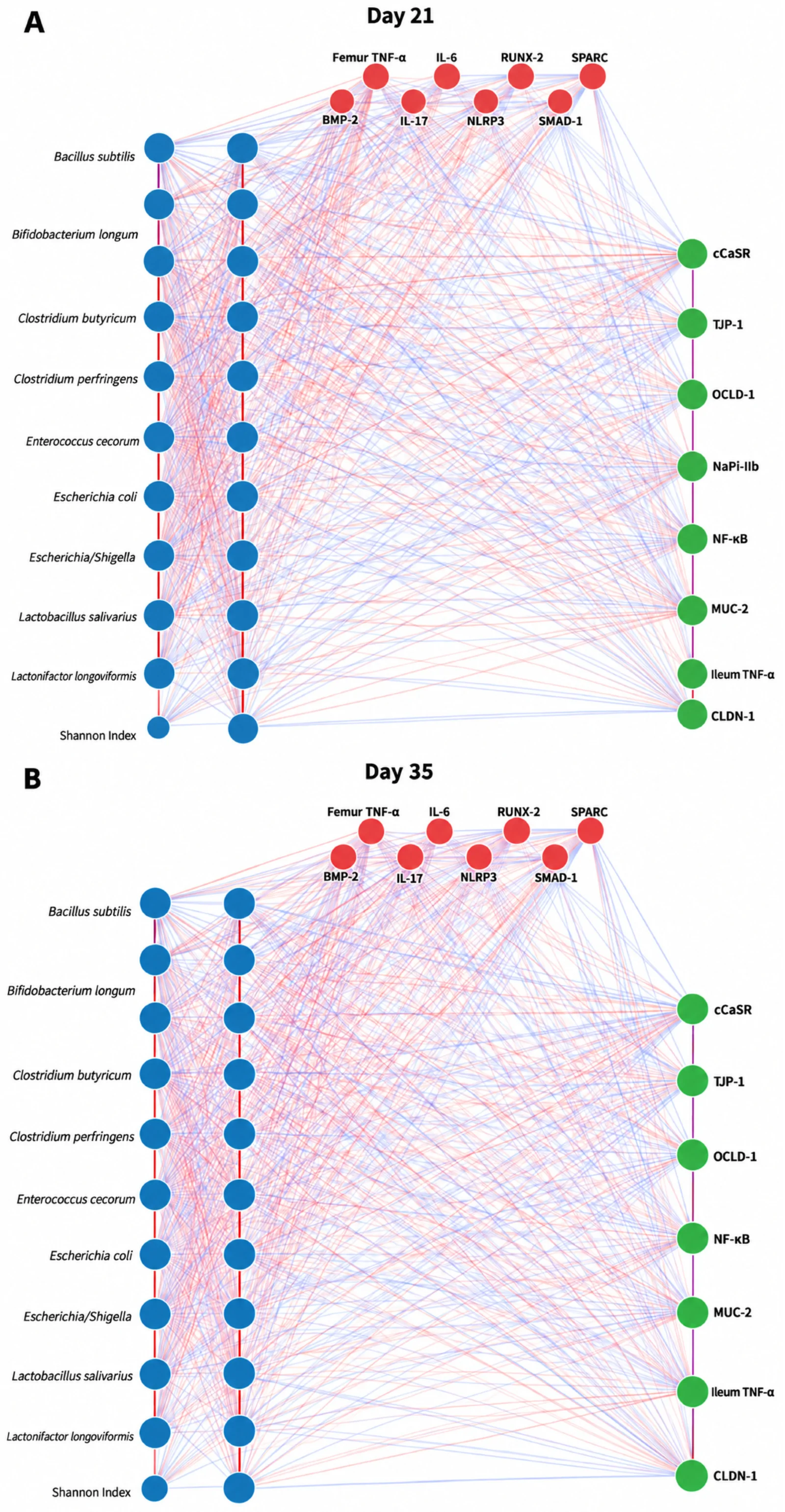

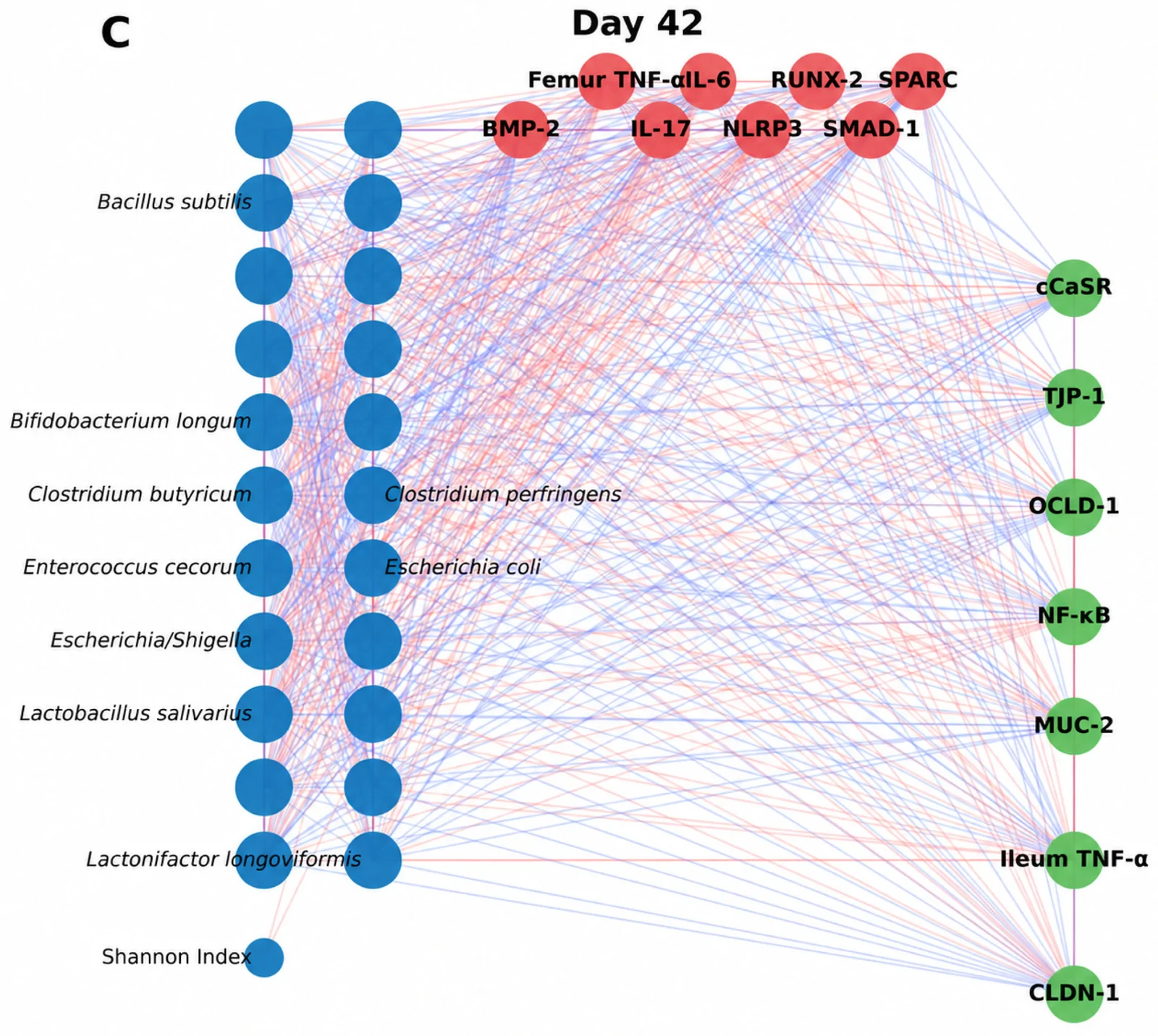
(**A**) Exploratory microbiome–gut–bone association network at day 21. Native source artwork is shown at an enlarged size without changing nodes or edges. Blue, green, and red nodes denote the source microbial, intestinal, and femoral groups. Blue and red edges denote positive and negative correlations; the original retention rule was |ρ| ≥ 0.50 and *p* < 0.05. (**B**) Exploratory microbiome–gut–bone association network at day 35. Native source artwork is shown at an enlarged size without changing nodes or edges. Blue, green, and red nodes denote the source microbial, intestinal, and femoral groups. Blue and red edges denote positive and negative correlations; the original retention rule was |ρ| ≥ 0.50 and *p* < 0.05. (**C**) Exploratory microbiome–gut–bone association network at day 42. Native source artwork is shown at an enlarged size without changing nodes or edges. Blue, green, and red nodes denote the source microbial, intestinal, and femoral groups. Blue and red edges denote positive and negative correlations; the original retention rule was |ρ| ≥ 0.50 and *p* < 0.05.

Age-stratified Spearman networks described associations among cecal microbial features, intestinal markers, and femoral inflammatory and osteogenic markers (Figure 9A–C). At day 21, the network contained 42 nodes and 721 retained associations (density = 0.837); 50.9% of edges were positive and 49.1% were negative. At day 35, the network contained 38 nodes and 639 associations (density = 0.909); 50.2% were positive and 49.8% were negative. The supplied day-42 graph contained 38 nodes and 640 unique associations (density = 0.910), comprising 321 positive (50.16%) and 319 negative (49.84%) edges. The original day-21 and day-35 summaries reported 361 and 367 cross-domain associations, respectively. These descriptors summarize the constructed graphs; they do not demonstrate statistically tested temporal reorganization, shared-edge stability, or causal relationships.

### 3.9. Clinical Lameness Observations

Clinical lameness was recorded in six distinct birds in the control group and four distinct birds in the BsP group during the observation period. Each affected bird was counted once. These counts are reported descriptively and do not constitute confirmed BCO diagnoses because lesions were not systematically assessed. All birds selected for laboratory analyses were clinically normal at sampling.

## 4. Discussion

The combined BsP diet was associated with improved final growth and feed conversion together with intestinal, microbial, mineral, and femoral responses. These findings extend reports that *B. subtilis* DSM 29784 can alter broiler performance and intestinal biology [14,26,36]. Because only the combined intervention was tested, the individual contributions of the probiotic and phytogenic blend and any synergistic interaction cannot be resolved.

### 4.1. Intestinal Barrier, Inflammation, and Mineral-Transport Responses

The intestinal findings involve distinct components of epithelial defense. *CLDN-1* and *OCLD-1* are tight junction components, whereas *TJP-1* provides a junctional scaffold; higher expression is consistent with altered capacity for junction maintenance. *MUC-2* contributes to the mucus barrier rather than constituting a tight junction protein. Its increase therefore represents a complementary mucus-associated response. These interpretations are consistent with intestinal responses reported for *B. subtilis* DSM 29784 in broilers [26] and with *MUC-2* and permeability responses in in vitro models exposed to its metabolites [36]. Lower ileal *TNF-α* and *NF-κB* transcript levels suggest reduced inflammatory transcription, potentially relevant to intestinal homeostasis [12,37]. However, transcript abundance does not demonstrate protein localization, functional permeability, or *NF-κB* activation; those endpoints were not directly measured here.

The mineral-related markers also have different roles: *cCaSR* senses extracellular calcium and participates in homeostatic signaling, whereas *NaPi-IIb* mediates sodium-dependent intestinal phosphate transport [38,39]. Their higher expression alongside increased serum and femoral mineral concentrations at days 21 and 35 is compatible with a coordinated mineral-related response, but it does not establish greater absorption or whole-body retention. A broiler study of *Enterococcus faecium* linked *NaPi-IIb* expression with direct phosphorus utilization measurements [40]; such functional measurements would be needed to test the corresponding interpretation for BsP.

### 4.2. Cecal Microbiome and Predicted Functional Potential

The cecal response is consistent with the sensitivity of chicken microbial communities to age and diet [41,42] and with reported effects of probiotics on intestinal ecology [14,15,43,44,45,46,47,48]. Treatment-associated taxonomic differences can identify candidate ecological relationships, but relative abundance and greater diversity do not independently establish microbial activity, absolute bacterial load, or improved health. The findings should therefore be interpreted together with the measured host responses rather than as proof that individual taxa mediated the performance benefit.

PICRUSt2 estimates microbial functional potential from marker gene profiles [29]. Accordingly, the tryptophan-, serotonin-, melatonin-, phosphate-, and glutamate-related results describe predicted pathway representation, not measured synthesis, secretion, or metabolic flux. Circulating serotonin was measured by HPLC in this study, but its concentration does not identify its microbial source or validate the predicted pathways. Direct cecal measurements of short-chain fatty acids, serotonin, melatonin, and indole derivatives, supported by metagenomic or metatranscriptomic analysis, are appropriate next steps for testing these candidate mechanisms.

### 4.3. Osteoimmunological and Femoral Responses

The skeletal evidence was not restricted to gene expression: femoral calcium and phosphate concentrations were also measured (Table 4). Inflammatory and osteogenic transcripts, together with mineral concentrations, describe molecular and compositional responses rather than functional bone quality. *BMP/SMAD/RUNX2* signaling and *SPARC* are relevant to osteogenic processes [6,49,50], but their expression does not quantify bone formation rate or mechanical resistance. Thus, the higher mineral concentrations at days 21 and 35 provide complementary evidence, not proof of stronger bones. Mechanical testing and systematic clinical and lesion assessment are required to determine whether these biological responses translate into skeletal protection.

### 4.4. Femoral Microbial DNA Signatures and BCO-Associated Bacteria

Lower femoral dPCR signals for selected BCO-associated bacteria are relevant to hypotheses about intestinal reservoirs and skeletal exposure [47,51,52,53,54,55,56], but DNA detection does not establish viability, bacterial translocation, or disease. Femoral 16S profiles also arise from a low-biomass matrix and remain exploratory bacterial DNA signatures without the dedicated contamination controls or orthogonal confirmation described as limitations in the original workflow. The targeted dPCR results provide complementary molecular observations, not confirmed BCO diagnoses. *Bacillus* strains can inhibit avian *Enterococcus cecorum* isolates in vitro, but these effects are strain- and isolate-dependent [47]. This provides a rationale for mechanistic follow-up, not evidence that *B. subtilis* DSM 29784 or the phytogenic blend independently prevented infection in the present combined-intervention experiment.

### 4.5. Age-Dependent Microbiome–Gut–Bone Network Organization

The age-specific networks summarize covariation rather than developmental trajectories in individual birds. Day-35 and day-42 graphs had similar overall node counts and densities, but this does not demonstrate that the same associations were retained. Differences from day 21 remain descriptive because the node sets differed and different birds were sampled at successive ages. Dense graphs can reflect shared treatment responses and the number of tested pairs as well as biological relationships. The networks are therefore useful for nominating candidate associations, not identifying causal hubs or establishing mediation. Separate high-resolution panels and the day-42 edge/node files improve accessibility without selectively deleting retained associations.

### 4.6. Study Limitations and Translational Implications

The combined-only design does not resolve component-specific effects or synergy. Predicted microbial functions require direct functional validation, and the exploratory networks and low-biomass femoral DNA profiles require cautious interpretation. Laboratory analyses were restricted to clinically normal birds, so they do not characterize treatment responses in birds with established disease. Lameness was observed in six control and four BsP birds, but these counts were reported descriptively; BCO lesions were not systematically assessed. Consequently, the study supports further evaluation of biological potential rather than a claim of preventive or therapeutic efficacy against BCO. Future factorial trials should combine direct bone quality measurements, standardized lameness and lesion assessments, and targeted microbial metabolite measurements.

## 5. Conclusions

Combined supplementation with *B. subtilis* DSM 29784 and a phytogenic blend improved final growth and overall feed conversion and was associated with changes in intestinal and femoral gene expression, circulating and femoral mineral concentrations at selected ages, cecal microbial profiles, and targeted bacterial DNA signals. The findings concern microbial, molecular, and mineral responses in clinically normal sampled birds, not demonstrated mechanical bone improvement or BCO prevention. PICRUSt2 predictions and age-specific networks provide hypotheses for further testing. Factorial studies with direct bone quality and clinical outcomes and targeted metabolite measurements are needed to determine component-specific effects and preventive potential.

## Figures and Tables

**Table 1:** Ingredients and nutrient composition of the basal experimental diets (as-fed basis).

Items	Starter (1 to 21 d)	Grower (22 to 35 d)	Finisher (36 to 42 d)
Ingredient, (%)			
Corn	53.18	57.43	60.05
Soybean Meal	30.25	24.90	21.70
Full Fat Soybean	8.00	9.00	10.00
Soybean Oil	4.27	4.70	4.45
MCP	1.40	1.27	1.19
Calcium Carbonate	1.15	1.00	0.96
Pellet Binder	0.30	0.30	0.30
Salt	0.26	0.26	0.26
^1/^ Broiler Vit/Min Premix	0.20	0.20	0.20
DL-Methionine	0.31	0.28	0.26
L-Lysine HCl	0.13	0.13	0.12
L-Threonine	0.12	0.10	0.09
Sodium Bicarbonate	0.14	0.14	0.14
Choline Chloride 60%	0.08	0.08	0.07
Antimold	0.20	0.20	0.20
Antioxidant	0.01	0.01	0.01
Nutrient Composition			
Metabolizable Energy, kcal/kg	3100	3180	3200
Protein (%)	22.00	20.00	19.00
Moisture (%)	11.50	11.65	11.55
Fat (%)	6.40	6.92	6.99
Fiber (%)	3.40	3.66	3.56
Ash (%)	6.68	6.61	6.55
Ca (%)	0.89	0.80	0.76
Total P (%)	0.71	0.66	0.64
P Avail (%)	0.44	0.40	0.38
Salt by Na (%)	0.16	0.16	0.16
Dig Lysine (%)	1.18	1.05	0.99
Dig Methionine (%)	0.60	0.55	0.52
Dig Met + Cys (%)	0.89	0.82	0.77
Dig Cysteine (%)	0.29	0.27	0.26
Choline (mg/kg)	1700	1600	1500

^1/^ Broiler trace mineral and vitamin premixes added at this rate yields (per kg of diet); 100 g of manganese, 100 mg of zinc, 40 mg of iron, 15 mg of copper, 1.00 mg of iodine, 0.30 mg of selenium, 12,000,000 IU of vitamin A, 2,400,000 IU of vitamin D_3_, 60 mg of vitamin E, 0.020 mg of B_12_, 8.00 mg of riboflavin, 50 mg of niacin, 15 mg of D-pentothenic acid, 3.00 mg of menadione, 2.00 mg of folic acid, 4.00 mg of peroxide, 3.00 mg of thiamine, and 0.40 mg of biotin. The carrier is ground rice hulls.

**Table 2:** Primer used for qPCR amplification of target genes of gut integrity, inflammatory cytokines, calcium phosphate receptor, and osteoblast activity.

Gene	Biomarker	Sample	GenBank Gene Accession No.	Primer Sequence (5′-3′)	Size (bp)	Annealing Temp.
*CLADN1*	Gut integrity: Tight junction	Jejunum	NM_001013611.2	F:GGCCACGTCATGGTATGG R:AACGGGTGTGAAAGGGTCATAG	61	60
*OCLD*	Gut integrity: Tight junction	Jejunum	NM_205128.1	F:ACGGCAGCACCTACCTCAA R:GGGCGAAGAAGCAGATGAG	123	60
*TJP-1*	Gut integrity: Tight junction	Jejunum	XM_040680630.2	F:GGATGTTTATTTGGGCGGC R:GTCACCGTGTGTTGTTCCCAT	187	60
*MUC-2*	Gut integrity: Mucus barrier	Jejunum	XM_040673077.2	F:CCCTGGAAGTAGAGGTGACTG R:TGACAAGCCATTGAAGGACA	143	60
*IL-6*	Inflammatory	Femur	NM_204628.2	F:CAGCTGCAGGACGAGATGTGCAA R:GCACAGAGACTCGACGTTCTGCT	237	60
*IL-17*	Inflammatory	Femur	NM_204460.2	F:TATCAGCAAACGCTCACTGG R:AGTTCACGCACCTGGAATG	110	60
*NLRP-3*	Inflammatory	Femur	NM_001348947.2	F:CTGAAGTGCCTGGACCTGAGT R:TGTAGAAGTGCTCAGCCCCAG	114	60
*TNF-α*	Inflammatory	Femur Ileum	NM_204267.2	F:GAGCGTTGACTTGGCTGTC R:AAGCAACAACCAGCTATGCAC	64	60
*Nf-* *κβ*	Inflammatory	Ileum	NM_205129	F:GTGTGAAGAAACGGGAACTG R:GGCACGGTTGTCATAGATGG	143	58
*NaPi-IIb*	Phosphate receptor	Ileum	NM_204474.1	F:CTTTTACTTGGCTGGCTGGAT R:AGGGTGAGGGGATAAGAACG	148	60
*cCaSR*	Calcium receptor	Ileum	XM_416491.6	F:CAAACCAACGGGCACAGAAG R:ATGCACTCCACTGATTCGGG	64	60
*BMP-2*	Osteoblast activity	Femur	NM_001398170.1	F:AGCTTCCACCACGAAGAAGT R:GGTAGCTGCTGTTGCTCTCA	172	60
*SMAD-1*	Osteoblast activity	Femur	NM_001201455.2	F:GTTTTGTAAAGGGTTGGGGAGC R:AATGCAGGAGCTTGGGACCTTA	174	60
*SPARC*	Osteoblast activity	Femur	NM_204410.2	F:GTATGAGCGCGATGAGGACA R:GTGGGACAGGTACCCATCAA	214	60
*RUNX2*	Osteoblast activity	Femur	NM_204128.1	F:GATTACAGACCCCAGGCAGG R:TGGCTCAAGTAGGACGGGTA	75	60
*Escherichia coli*	BCO bacterial	Cecum Femur	CP041623.1	F:TTCGTTTGCCGATTCACATC R:TCTCTTCCGCACTTGGTTCA	99	55
*Staphylococcus aureus*	BCO bacterial	Cecum Femur	CP118830.1	F:GCGATTGATGGTGATACGGTT R:AGCCAAGCCTTGACGAACTAAAGC	279	55
*Enterococcus cecorum*	BCO bacterial	Cecum Femur	MK308719.1	F:AAACATCATAAAACCTATTTA R:AATGGTGAATCTTGGTTCGCA	371	55
*GAPDH*	Housekeeping genes	All samples	NM_204305.2	F:TGACAAGTCCCTGAAAATTGTCA R:CAAGGGTGCCAGGCAGTT	61	60

Occluding (*OCLD*), claudin-1 (*CLDN1*), tight junction proteins-1 (*TJP-1*), mucin-2 (*MUC-2*), interleukin-6 (*IL-6*), interleukin-17 (*IL-17*), tumor necrosis factor alpha (*TNF-α*), *NLR* family pyrin domain-containing *3* (NLRP3) inflammasome, sodium phosphate type_IIb (*NaPi-IIb*), chicken calcium-sensing receptor (*cCaSR*), nuclear factor-κβ (*Nf-κβ*), bone morphogenetic protein-2 (*BMP-2*), osteonectin (*SPARC*), SMAD family member 1 (*SMAD-1*), and related transcription factor Runt 2 (*RUNX2*), and glyceraldehyde-3-phosphate dehydrogenase (*GAPDH*).

**Table 3:** Growth performances of broiler chickens during 1 to 42 days of supplemented *Bacillus subtilis* DSM 29784 combined with blended botanicals (BsP) compared with a basal diet (Ctrl).

Parameters	Ctrl	BsP	*p*-Value	Pool SE
Initial weight	43.08	42.92	0.612	0.150
1–7 d				
Body weight (g)	168.91	168.69	0.884	0.884
Body weight gain (g)	125.83	125.77	0.970	0.833
Feed intake (g)	167.62	167.37	0.903	1.033
FCR	1.332	1.331	0.905	0.008
8–14 d				
Body weight (g)	451.95	451.30	0.928	4.734
Body weight gain (g)	283.04	282.61	0.953	4.338
Feed intake (g)	357.22	352.09	0.569	5.147
FCR	1.262	1.246	0.037	0.006
15–21 d				
Body weight (g)	983.94	983.75	0.981	6.847
Body weight gain (g)	531.99	532.45	0.941	3.372
Feed intake (g)	646.04	644.76	0.887	4.038
FCR	1.214	1.211	0.714	0.008
22–28 d				
Body weight (g)	1695.71	1697.20	0.901	11.572
Body weight gain (g)	711.78	713.45	0.832	8.022
Feed intake (g)	1103.29	1105.15	0.829	5.605
FCR	1.550	1.549	0.882	0.014
29–35 d				
Body weight (g)	2505.19	2525.34	0.410	15.324
Body weight gain (g)	809.47	828.14	0.295	8.947
Feed intake (g)	1413.33	1428.54	0.861	8.571
FCR	1.746	1.725	0.003	0.289
36–42 d				
Body weight (g)	3252.39	3331.52	0.018	22.563
Body weight gain (g)	747.20	806.18	0.024	14.328
Feed intake (g)	1511.77	1557.10	0.256	18.514
FCR	2.023	1.931	0.038	0.218
1–42 d				
Body weight (g)	3252.39	3331.52	0.018	22.563
Body weight gain (g)	3209.31	3288.61	0.017	22.517
Feed intake (g)	5199.27	5255.01	0.221	21.772
FCR	1.620	1.597	0.017	0.110
Mortality (%)	2.542	2.783	0.987	0.728

Means represent eight replicates with 35 chicks per replicate for each treatment.

**Table 4:** Blood hormones and blood–femoral mineral concentrations of broilers during 21, 35, and 42 days of supplementation with *Bacillus subtilis* DSM 29784 combined with blended botanicals (BsP) compared with a basal diet (Ctrl).

Parameters	Day 21				Day 35				Day 42			
	Ctrl	BsP	S.E.	*p*	Ctrl	BsP	S.E.	*p*	Ctrl	BsP	S.E.	*p*
Blood chemical												
Serotonin, ng/mL	30.07	35.70	0.80	0.0009	33.40	38.60	0.56	0.0007	34.60	40.64	0.80	0.0010
Corticosterone, ng/mL	13.47	10.43	0.58	0.0060	16.69	13.73	0.80	0.0200	17.44	16.03	0.84	0.0300
Calcium (Ca), mg/dL	25.77	34.90	0.80	0.0003	21.79	28.90	0.84	0.0010	18.52	20.44	1.21	0.1800
Phosphate (Pi), mg/dL	8.47	9.88	0.44	0.0300	5.74	8.81	0.22	0.0001	3.77	4.12	0.13	0.0500
Femoral mineral												
Calcium (Ca), mg/g	151.40	205.90	2.82	0.0004	173.90	194.40	4.45	0.0100	196.60	185.70	32.92	0.7500
Phosphate (Pi), mg/g	64.41	83.79	1.25	0.0001	72.37	94.94	1.61	0.0001	107.60	110.50	3.06	0.3900

Values were least square mean; n is equal to 8 from each group.

## Data Availability

The raw 16S rRNA gene sequencing data from this study was deposited in the NCBI Sequence Read Archive (SRA). BioProject accession: PRJNA1541423.
